# Potential Use for Serosurveillance of Feral Swine to Map Risk for Anthrax Exposure, Texas, USA

**DOI:** 10.3201/eid2712.211482

**Published:** 2021-12

**Authors:** Rachel M. Maison, Courtney F. Pierce, Izabela K. Ragan, Vienna R. Brown, Michael J. Bodenchuk, Richard A. Bowen, Angela M. Bosco-Lauth

**Affiliations:** Colorado State University, Fort Collins, Colorado, USA (R.M. Maison, I.K. Ragan, R.A. Bowen, A.M. Bosco-Lauth);; US Department of Agriculture National Wildlife Research Center, Fort Collins (C.F. Pierce);; US Department of Agriculture National Feral Swine Damage Management Program, Fort Collins (V.R. Brown);; US Department of Agriculture Animal and Plant Health Inspection Service, San Antonio, Texas, USA (M.J. Bodenchuk)

**Keywords:** anthrax, Bacillus anthracis, bacteria, biosentinels, ELISA, endemic diseases, enzyme-linked immunosorbent assay, feral swine, invasive species, phylogeny, public health surveillance, risk assessment, serosurveillance, Sus scrofa, Texas, southern United States, zoonoses

## Abstract

Anthrax is a disease of concern in many mammals, including humans. Management primarily consists of prevention through vaccination and tracking clinical-level observations because environmental isolation is laborious and bacterial distribution across large geographic areas difficult to confirm. Feral swine (*Sus scrofa*) are an invasive species with an extensive range in the southern United States that rarely succumbs to anthrax. We present evidence that feral swine might serve as biosentinels based on comparative seroprevalence in swine from historically defined anthrax-endemic and non–anthrax-endemic regions of Texas. Overall seropositivity was 43.7% (n = 478), and logistic regression revealed county endemicity status, age-class, sex, latitude, and longitude were informative for predicting antibody status. However, of these covariates, only latitude was statistically significant (β = –0.153, p = 0.047). These results suggests anthrax exposure in swine, when paired with continuous location data, could serve as a proxy for bacterial presence in specific areas.

Anthrax, caused by *Bacillus anthracis*, is a zoonotic disease of global importance because of its ecologic effects on wildlife and free-ranging livestock and resulting economic impact on farmers and herders, its worldwide distribution, and its ability to cause disease even after decades of lying dormant in the environment. Known risks of exposure, considered together with unconfirmed environmental distribution in most regions and unidentified or evolving epidemiologic risk factors, make *B. anthracis* a pathogen of continuing human and animal health concern. 

*B. anthracis* is a gram-positive, endospore-forming bacterium. Anthrax cases have been clinically described since the 1700s, but symptomatic descriptions of the disease have been recorded as early as 1000 BCE (*1*,*2*). Genetic studies however, suggest that the geographic origin of *B. anthracis* was in sub-Saharan Africa; subsequent environmental spread followed the migration of humans and domesticated animals (*3*,*4*). Current case report data indicate that enzootic anthrax correlates with warmer climates, although some cases have been documented above the arctic circle, in Canada, and in northern Siberia (*5*). The true incidence of the disease remains unknown in many countries, although it is assumed that the bacterium resides in most regions (*6*). Extensive ecologic modeling efforts now offer some ability to predict outbreak risks spatially and temporally in several countries (*7*–*10*). Of note, recent modeling efforts have indicated that, in the United States, landscapes most capable of supporting *B. anthracis* span a north–south corridor encompassing most of the central United States and southwestern Texas (*11*).

Thought to affect all mammals to varying degrees, *B. anthracis* infection generally causes the highest levels of illness and death in herbivorous species (*12*,*13*). Exposure most commonly occurs when an animal ingests the dormant spore form of the bacterium, but cutaneous and inhalational infections also occur (*14*). Once inside a susceptible host, bacteria transform into a vegetative form that secretes a combination of lethal and edema factor proteins as well as the cell receptor–binding protein-protective antigen (PA), which mediates their entry into host cells and activates them to produce lethal factor and edema factor toxins, contributing to the ultimate death of susceptible hosts. Upon host death, exposure of vegetative bacilli to atmospheric oxygen, typically through carcass manipulation by scavengers, initiates the sporulation process, in which bacteria return to their dormant form. Sporulated *B. anthracis* is highly resistant to environmental degradation; some environmental isolations have detected viable spores up to 200 years old (*4*). Humans and other animals that encounter infected carcasses or animal materials are therefore at increased risk of exposure because infected carcasses that are manipulated or opened can initiate sporulation and consequently perpetuate the environmental persistence of infectious *B. anthracis*.

Current preventive management for domestic herbivores is primarily vaccine-based (*12*), but vaccination is not a requirement for livestock owners, who instead commonly use it reactively to control outbreaks (*11*,*15*). Outbreaks of anthrax in wild and domestic animals today are defined by the detection of carcasses, often from otherwise healthy animals. Unlike among domestic populations however, observation of anthrax is extremely difficult among wild or free-ranging herbivores, because detecting carcasses over large landscapes is an imperfect and likely inaccurate method for reporting true incidence, and wildlife usually cannot be observed for clinical signs of disease (*16*–*18*).

Humans, suids, and carnivores are considered incidental hosts and considerably less susceptible to lethal infection than herbivores (*19*). Although the causes of these variations in susceptibility remain largely unknown, it is likely they are a combination of differences in physiology, behavior, dosage, and transmission routes (*20*). For example, carnivores, omnivores, and scavengers all have lower stomach pH than herbivores, likely killing *B. anthracis* spores or vegetative cells incidentally ingested while foraging (*12*,*21*). In addition, some evidence indicates that necrophilic and hemophagic arthropods can contribute to infection (*19*,*22*), suggesting that transmission routes might also differ by a regions’ competent vector species. In endemic regions such as Africa, there appears to be little evidence of predators and scavengers dying of anthrax; those animals instead exhibit a high prevalence of antibodies against the bacterium (*20*). On the basis of these observations, it has previously been proposed that anthrax-resistant suid species, such as the Eurasian wild boar (*Sus scrofa*) in Ukraine and feral hog in the United States, might be used as biosentinels for anthrax (*23*). Of note, although a previous study (*23*) described serologic evidence of exposure in wild boars Ukraine, no studies to date have formally evaluated exposure in taxonomically identical feral swine (also *S. scrofa*) present in the United States. Introduced initially in the 1500s to states bordering the Gulf of Mexico, populations of feral swine have exploded since the 1980s and have become established throughout most suitable habitats in the southern United States (*24*).

In addition to known pathways of transmission, the shared presence of *B. anthracis* and anthrax-resistant wildlife species might contribute to anthrax epidemiology under certain conditions by increasing the risk for exposure to humans or more susceptible herbivorous species. Resistant species may also help to disseminate infectious spores to new landscapes through mechanical transmission or bacterial shedding (*6*,*25*). Feral swine are known to be opportunistic omnivores that occasionally scavenge carcasses, as well as routinely root in soils for food (*26*). These behaviors, coupled with their documented resistance to anthrax, suggest that feral swine might be a good indicator of bacterial presence on the landscapes they occupy. We report the potential biosentinel utility of feral swine for measuring anthrax distribution by examining antibody prevalence in confirmed endemic and nonendemic regions of Texas, USA.

## Materials and Methods

### Study Area

We conducted our investigation in Texas because anthrax is a reportable disease and is relatively predictable in select regions of the state. Feral swine populations are also present in most counties, offering a unique opportunity to evaluate the species as a biosentinel for *B. anthracis*. In addition, observations by residents of the state’s endemic region have described resurgences in anthrax in areas recently colonized by feral swine, anecdotally suggesting the 2 events might be related.

**Figure 1:**
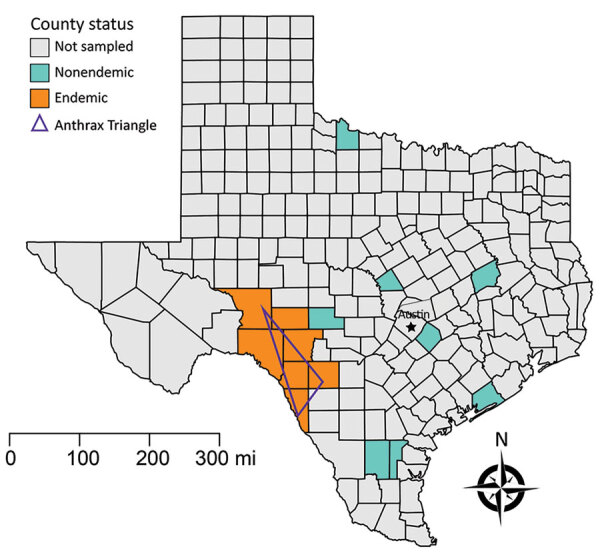
Field sampling designations for feral swine serum samples collected in Texas, USA. The Anthrax Triangle designates a region that experiences semiregular outbreaks of anthrax in both domestic and wildlife species. All other Texas counties are considered nonendemic, but we serosampled only 7 of those counties.

Outbreaks of anthrax occur regularly in portions of Crockett, Val Verde, Sutton, Edwards, Kinney, Uvalde, and Maverick Counties, colloquially referred to as the Anthrax Triangle, usually in dry summer months following heavy spring rains (*27*,*28*). Conversely, eastern Texas does not experience regular outbreaks, despite also being heavily populated with domestic livestock (*29*). Furthermore, populations of ranched white-tailed deer in areas of Val Verde, Uvalde, and Webb Counties are also regularly affected, suggesting wild herbivores in the same region might become infected at similar rates. We binarily defined areas as either endemic for anthrax for those 7 counties on the western side of the state comprising the historic Anthrax Triangle (Figure 1) or nonendemic if outside of this region, because these counties do not experience regular, seasonal cases.

### Field Sampling

Wildlife Services, a branch within the US Department of Agriculture (USDA), routinely removes feral swine from the landscape for damage control and invasive species management, and as part of these efforts, collect serum samples from a subset of swine for disease surveillance. Samples not used for routine surveillance are archived and can be used for select retrospective studies. Through these efforts, we obtained 478 serum samples collected during 2007–2019 from feral swine removed from areas throughout Texas and tested them to determine the prevalence of feral swine exposure to *B. anthracis* by measuring antibodies against PA. We illustrated spatial data on the geographic origins of the feral swine serum samples (Table 1; Figure 1) at the county level to protect personally identifiable information because many samples were collected on private property. Approximately half (n = 243) of the serum samples originated in the 7 endemic counties within the Anthrax Triangle and the rest (n = 235) from 7 nonendemic counties outside of it. We randomly selected the 7 nonendemic counties from the 246 Texas counties located outside of the Anthrax Triangle; 7 counties were selected so that the sampling effort was equal between endemic and nonendemic regions. Sampling events took place year-round.

**Table 1:** Sampling distribution of feral swine serum samples collected from endemic and nonendemic regions of Texas, USA.

Region type	Male			Female		Total
	Adult	Subadult		Adult	Subadult	
Endemic	90	16		121	16	243
Nonendemic	113	12		101	9	235
Total	203	28		222	25	478

Serum samples were taken from male and female feral swine classified as either adult, estimated by Wildlife Services field personnel to be >1 year of age, or subadult, estimated as 2 months–1 year of age (Table 1). We did not collect samples from juveniles (<2 months of age) to avoid confounding serology that could result from the presence of maternal antibodies (*30*). All blood samples were collected postmortem and serum extracted within 12 hours of clotting and shipped overnight on ice to the National Wildlife Research Center (Fort Collins, CO, USA), where they were stored at –80°C until testing.

### Serology

We used an indirect ELISA platform similar to those described elsewhere (*31*–*34*), with slight modifications to target antibodies of swine origin. We assayed samples blindly relative to the origin, sex, and age-class of individual animals until all results were finalized. We coated high binding polystyrene 96-well flat-bottom microtiter plates (ThermoFisher Scientific, https://www.thermofisher.com) with recombinant protective antigen (rPA) from *B. anthracis* (American Type Culture Collection, https://www.atcc.org) diluted in carbonate buffer solution at a concentration of 5 μg/mL per well and incubated plates overnight at 4°C. The following day, we discarded the coating buffer and washed the wells 5× with phosphate-buffered saline containing 0.05% Tween 20 washing buffer. We blocked wells by adding 300 μL of 10% skim milk in phosphate-buffered saline and allowed plates to incubate for 1.5 h at room temperature. We again washed wells, then added 100 µL of test serum diluted 1:100 in blocking buffer and incubated plates for 1 h with shaking at room temperature. After additional washing, we added 100 µL/well of protein A/G-horseradish peroxidase (ThermoFisher Scientific) diluted 1:1,000 in blocking buffer, and further incubated plates with shaking for 30 min. After 1 final washing step, we added 150 µL of one-step ABTS (ThermoFisher Scientific), incubated for 15 min, and then added 100 µL of 1% sodium dodecyl sulfate solution to stop the reaction. We measured absorbance at 25°C and 405 nm using a BioTek microplate reader paired with Gen5 version 3.09 microplate reader and imager software (https://www.biotek.com). We considered samples positive for rPA antibodies if their mean absorbance measurements were >3 times the SD above the mean of the negative controls. We ran individual samples in triplicate.

Because of their inherent resistance to anthrax infection, domestic pigs are not as routinely vaccinated as ruminant livestock species. As such, swine serum samples were unavailable for use as antibody-positive and -negative controls for this assay. Instead, we obtained control serum samples included in each assay from one male domestic goat (*Capra aegagrus hircus*) before and after vaccination with Anthrax Vaccine Adsorbed (BioThrax, https://www.beiresources.org). Protein A/G is known to bind to the constant region of both goat and swine IgG with comparable affinity (*35*–*37*).

### Statistics

We examined how the probability of an individual animal being positive for anthrax antibodies varied by region (endemic vs. nonendemic), sex, age-class (adult vs. subadult), latitude, and longitude using logistic regression and mixed-effects models implemented in R version 4.0.2 (R Foundation for Statistical Computing, https://www.r-project.org). We examined region, sex, age-class, latitude, and longitude as fixed effects and evaluated sampling year as a random effect to account for temporal variation in anthrax prevalence and sampling. Since most anthrax cases in Texas originate from the Anthrax Triangle (*27*,*28*), we included region as a fixed effect to evaluate whether feral swine residing in known contaminated environments are more likely to be antibody positive than those outside. We used county centroids as a proxy for sampling locations and considered latitude and longitude fixed effects to account for spatial trends in anthrax prevalence. Interaction between age-class and sex was also examined to account for potential impacts of age variations by sex.

We evaluated support including a random effect (sampling year) using Akaike’s information criterion (AIC) and likelihood ratio test (LRT) in R. As recommended elsewhere (*38*), we first examined whether sample year should be included by comparing AIC and LRT with and without its addition from a fully parameterized fixed effects model. If the random effect was supported (ΔAIC >2 compared with the model excluding the random effect), then it was retained in all models and the fixed effects compared. Using LRT as an additional method of evaluating the inclusion of sampling year, we calculated the difference in the log likelihoods of the 2 nested models (i.e., fully parameterized fixed effect model with or without the addition of the random effect) and if the difference was statistically significant (α = 0.05), we included the random effect in all models. 

We compared all combinations of fixed effects covariates using AIC implemented in the R package MuMIn (R Foundation for Statistical Computing); the lowest AIC value represented the most parsimonious model. If model uncertainty existed (i.e., >1 competing model <2 ΔAIC of the top model), we examined the relative support for each covariate by calculating cumulative covariate weights; we considered weights >0.5 supported (*39*). We selected the final model based on the supported covariate regression coefficients used to calculate odds ratios and 95% CI for the probability of having anthrax antibodies by covariate. Finally, to assess model fit we calculated area under the curve (AUC) for the receiver operating characteristic (ROC) (*40*) curve using the pROC (partial receiver operating characteristic) curve package in R (*41*); the ROC curve enabled us to assess the performance of the binary classification model for identifying individual animals as positive or negative. To summarize the ROC curve, we calculated the AUC, an aggregated measure of binary classification model performance, in which the model AUC = 0.5 for no predictive power, >0.5–<0.7 for poor predictive power, ≥0.7–<0.8 for acceptable predictive power, and ≥0.8–<0.9 for excellent predictive power (*40*).

## Results

### Serology

Negative control goat serum collected before vaccination exhibited absorbance readings of 0.018–0.11 (mean 0.08, SD 0.022). Pooled positive serum taken 3 and 5 weeks after anthrax vaccination exhibited an absorbance range of 0.26–3.42 (mean 1.62, SD 1.27). We calculated the assay cutoff of +3 SD above the mean of the negative controls at 0.15. Of the 478 samples examined, we identified 209 (43.7%) as positive and 269 (56.3%) as negative for PA antibodies. From the entire sample pool, we recorded a minimum absorbance value of –0.006 and maximum value of 3.9.

### Statistics

**Figure 2:**
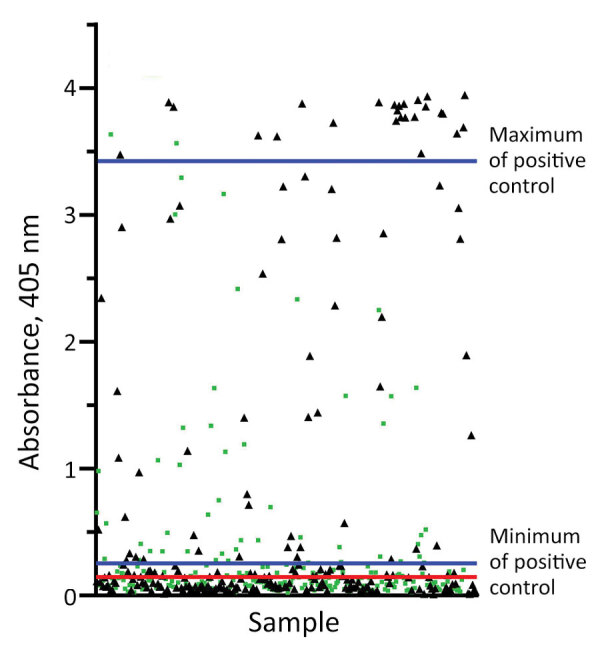
Sample absorbance values measured by ELISA at 405 nm for 478 feral swine serum samples collected from defined endemic and nonendemic regions of Texas, USA. The red cutoff line represents the calculated assay cutoff between seropositive and seronegative animals (e.g., +3 SD above the mean of the negative control), equal to 0.15 absorbance units. Blue lines delineate the absorbance unit range of the positive assay control. Black triangles represent samples taken in endemic counties; green boxes represent samples taken in nonendemic counties.

Basic data structure, including anthrax antibody status stratified by covariate and apparent seroprevalence (Table 2), includes raw data confirming that more swine from the endemic region (49.49%) compared with the nonendemic region (37.45%) were seropositive; we also illustrate individual sample absorbance by region (Figure 2). Seroprevalence was higher among female (48.18%) than male (38.96%) swine and among adult (44.71%) than subadult (35.85%) swine. The fully parametrized model failed to converge, so we excluded longitude and the interaction term (age-class*sex) from the fully parameterized model to evaluate inclusion of sampling year as a random effect. Sampling year did not improve the predictive power of the model (fixed effects model AIC = 649.87 and mixed-effects model AIC = 648.59; LRT p = 0.070); probability of an individual animal being seropositive was therefore best predicted by a fixed effects model. There was uncertainty about the optimal model (7 models were <2 ΔAIC). To determine their relative importance, we examined cumulative covariate weights and found that county endemicity status, age-class, sex, latitude, and longitude were informative for predicting antibody status, and therefore included them in the final model. We calculated odds ratios and 95% CI for each predictor variable (Table 3), but only latitude was statistically significant (β = –0.153; p = 0.047). The final model had poor predictive ability (AUC = 0.613) suggesting the presence of unexplained variance in anthrax antibody status.

**Table 2:** Distribution of anthrax seroprevalence in feral swine by region, sex, and age group.

Predictor	No. tested	No. positive	Apparent seroprevalence, %
Region			
Endemic	243	121	49.49
Nonendemic	235	88	37.45
Sex			
M	231	90	38.96
F	247	119	48.18
Age group			
Subadult	53	19	35.85
Adult	425	190	44.71

**Table 3:** Odds ratios and 95% CIs of the probability of having anthrax antibodies by fixed effects covariates

Covariate	Odds ratio (95% CI)
County status: endemic	1.035 (0.523–2.054)
Age class: adult	1.641 (0.903–3.059)
Sex: female	1.398 (0.966–2.026)
Latitude	0.858 (0.737–0.997)
Longitude	0.877 (0.702–1.092)

## Discussion

Serologic surveillance in various anthrax-resistant species has assisted wildlife managers and health officials in identifying areas of high outbreak risk (*20*) and the surprisingly high seroprevalence we identified in feral swine supports this strategy. *B. anthracis* spores exist in soil and the carcasses of animals that have died from anthrax, but the sampling efforts required to identify contaminated environments and subsequent outbreak risks are often too laborious or expensive to use, making the use of biosentinels an appealing option. In addition, human and animal case reports and mortality data likely underestimate the geographic extent of this pathogen, while exposure data obtained through serosurveillance might enable acquisition of multidimensional biologic information, such as environmental range and relative time of exposure. Because swine are resistant to anthrax (*19*) and there is serologic evidence of exposure in taxonomically identical species such as wild boar in Ukraine, feral swine might be good indicators of bacterial presence throughout their range in the United States. Feral swine also exhibit relatively small home ranges, 1–5 km^2^ (*41*,*42*), potentially enabling high resolution in estimating the geographic extent of contaminated environments.

Data presented here demonstrate that the overall odds of feral swine in Texas with anthrax antibodies differ between those inhabiting broadly defined endemic and nonendemic regions; animals originating within the Anthrax Triangle exhibit higher odds of being seropositive than those outside. This finding is not surprising given the regularity of outbreaks in domestic herbivores within this region and supports our preliminary hypothesis that feral swine are being exposed in regions experiencing regular occurrences of the disease. However, ≈37% of individual animals from nonendemic counties were also seropositive, so county status alone proved not to be a significant predictor covariate, and the size of that proportion suggests that bacteria might be present and therefore swine exposed beyond the confines of the Anthrax Triangle. This possibility is further supported by latitude but not county status being a statistically significant covariate in our top-performing model.

Although the role that feral swine might play in the overall epidemiology of anthrax is unknown, swine do exhibit close relationships with soil (*26*) and thus likely experience higher rates of exposure than humans and perhaps some domestic and wild ruminants; therefore, they might contribute to bacterial spread through biologic or mechanical dissemination. However, the level of exposure might simply reflect bacterial presence irrespective of swine involvement in dissemination, because outbreaks outside of the Anthrax Triangle are reported occasionally (*28*). Although our statistical analysis was unable to distinguish anthropogenically defined endemic and nonendemic regions, the high apparent seroprevalence observed in feral swine across the state of Texas is still useful information, because exposure data are further indicative of bacterial distribution occurring beyond the confines of the Anthrax Triangle, as has been predicted by the ecologic modeling efforts of others (*8*,*11*).

Of note, female and adult swine tended to have higher seropositivity than male and subadult swine, although the measures were not statistically significant. Higher odds by sex might be because of the inherent dynamics of swine sounders; groups typically are composed of several females and their offspring, whereas adult and subadult males are often solitary, only associating with females during breeding (*26*). The likelihood then of observing seropositive female swine in a *B. anthracis*–contaminated region might be higher simply because female swine traveling together are experiencing the same environmental exposures compared with their solitary male counterparts. The potential age-class bias observed could be explained in part by the unequal sample sizes between these covariates; more extensive data might be necessary to confirm this association. Finally, feral swine have been observed to opportunistically feed on carcasses of other animals, as well as prey on some livestock (*43*–*45*). Thus, feral swine might be contributing to anthrax epidemiology through a variety of mechanisms, including carrying and depositing spores or vegetative cells acquired from rooting in soil or by feeding on the carcasses of animals who have died from anthrax.

As with any retrospective, opportunistic serosurvey, the data and subsequent findings presented here are not without limitations. First, the fact that we broadly defined regions as endemic and nonendemic solely on the basis of whether a county was located in the Anthrax Triangle likely does not account for the contiguous or disjointed presence of this bacterium predicted in soils throughout the state (*11*), and counties that were sampled on the border of the Anthrax Triangle, such as Kimble, might have skewed results with some antibody-positive animals originating from this region. Also, in conjunction with the regions we defined, we did not examine any environmental conditions or weather patterns, which likely are substantial factors influencing bacterial distribution and infectivity rates between the sampling years examined and could be the source of the unexplained variance suggested during model evaluation.

In conclusion, feral swine are a fecund invasive species that often encounter people and domestic animals, as well as other wildlife species. Past investigations have identified myriad pathogens that can be transmitted or carried by these animals (*46*), and national programs supported by the USDA regularly survey populations for diseases of national concern to humans or related to agriculturally important species (*24*). Despite the amount of attention feral swine receive for harboring some pathogens, future investigations are needed to fully define the role feral swine play in anthrax epidemiology, particularly whether they are contributing to bacterial dissemination. However, our investigation suggests that levels of anthrax exposure in feral swine, when paired with continuous location data, could serve as a proxy for identifying *B. anthracis* presence in a specific area.
